# Healthcare professional views about a prehospital redirection pathway for stroke thrombectomy: a multiphase deductive qualitative study

**DOI:** 10.1136/emermed-2023-213350

**Published:** 2024-05-10

**Authors:** Jo Day, Rosemary L Simmonds, Lisa Shaw, Christopher I Price, Graham McClelland, Gary A Ford, Martin James, Phil White, Ken Stein, Catherine Pope

**Affiliations:** 1 NIHR Applied Research Collaboration South West Peninsula, Health and Community Sciences, University of Exeter, Exeter, Devon, UK; 2 Health and Community Sciences, University of Exeter, Exeter, Devon, UK; 3 Stroke Research Group, Population Health Sciences Institute, Newcastle University, Newcastle upon Tyne, UK; 4 North East Ambulance Service NHS Foundation Trust, Newcastle Upon Tyne, UK; 5 Northumbria University, Newcastle upon Tyne, Tyne and Wear, UK; 6 Oxford University Hospitals NHS Foundation Trust and Radcliffe Department of Medicine, University of Oxford, Oxford, UK; 7 Royal Devon University Healthcare NHS Foundation Trust and University of Exeter, University of Exeter, Exeter, Devon, UK; 8 Stroke Research Group, Clinical and Translational Research Institute, Newcastle University, Newcastle upon Tyne, UK; 9 NIHR Applied Research Collaboration South West Peninsula, University of Exeter, Exeter, Devon, UK; 10 Nuffield Department of Primary Care Health Sciences, University of Oxford, Oxford, UK

**Keywords:** emergency care systems, pre-hospital care, qualitative research, stroke

## Abstract

**Background:**

Mechanical thrombectomy for stroke is highly effective but time-critical. Delays are common because many patients require transfer between local hospitals and regional centres. A two-stage prehospital redirection pathway consisting of a simple ambulance screen followed by regional centre assessment to select patients for direct admission could optimise access. However, implementation might be challenged by the limited number of thrombectomy providers, a lack of prehospital diagnostic tests for selecting patients and whether finite resources can accommodate longer ambulance journeys plus greater central admissions. We undertook a three-phase, multiregional, qualitative study to obtain health professional views on the acceptability and feasibility of a new pathway.

**Methods:**

Online focus groups/semistructured interviews were undertaken designed to capture important contextual influences. We purposively sampled NHS staff in four regions of England. Anonymised interview transcripts underwent deductive thematic analysis guided by the NASSS (Non-adoption, Abandonment and Challenges to Scale-up, Spread and Sustainability, Implementation) Implementation Science framework.

**Results:**

Twenty-eight staff participated in 4 focus groups, 2 group interviews and 18 individual interviews across 4 Ambulance Trusts, 5 Hospital Trusts and 3 Integrated Stroke Delivery Networks (ISDNs). Five deductive themes were identified: (1) (suspected) stroke as a condition, (2) the pathway change, (3) the value participants placed on the proposed pathway, (4) the possible impact on NHS organisations/adopter systems and (5) the wider healthcare context. Participants perceived suspected stroke as a complex scenario. Most viewed the proposed new thrombectomy pathway as beneficial but potentially challenging to implement. Organisational concerns included staff shortages, increased workflow and bed capacity. Participants also reported wider socioeconomic issues impacting on their services contributing to concerns around the future implementation.

**Conclusions:**

Positive views from health professionals were expressed about the concept of a proposed pathway while raising key content and implementation challenges and useful ‘real-world’ issues for consideration.

**WHAT IS ALREADY KNOWN ON THIS TOPIC**

- Thrombectomy is a time-sensitive treatment that greatly improves chances of recovery from severe ischaemic stroke, but it is only available in a limited number of regional centres.
- Treatment is delayed for many people who must undergo secondary transfer to a regional centre from a local hospital.
- A new redirection pathway may improve this situation but before evaluation and implementation, health professionals’ views are required to understand acceptability, feasibility and potential real-world trade-offs/wider impact on systems and services.

**WHAT THIS STUDY ADDS**

- Most interviewed professionals found the concept of a pathway consisting of a simple ambulance screen and remote specialist assessment to be acceptable and feasible.
- Perceived logistical and resource challenges to implementing the pathway were identified.

**HOW THIS STUDY MIGHT AFFECT RESEARCH, PRACTICE OR POLICY**

- Policies supporting direct admission to specialist centres must acknowledge the complicated and complex nature of cross-organisational emergency care.
- Due to the resource and implementation challenges, further evidence is needed to understand how a direct admission pathway for thrombectomy will impact on the different patient groups and services involved.
- The use of the NASSS (Non-adoption, Abandonment and Challenges to Scale-up, Spread and Sustainability, Implementation) framework as a deductive framing device is useful for eliciting and organising important ‘real-world’ issues for consideration by those developing new emergency pathways.

## Introduction

Stroke is the most common cause of severe adult disability^1 2^ and the fourth largest cause of death in the UK.^3^ Outcomes can be significantly improved by emergency treatments.^4–8^ Thrombolysis within 4.5 hours of symptom onset has a number needed to treat of seven patients to avoid future dependency for one person.^5 6^ The addition of mechanical thrombectomy for 10–15% stroke patients with severe symptoms due to large artery occlusion (LAO) leads to a significantly better chance of recovery when performed within 6 hours (number needed to treat 2.6 patients), while recent trials show similar benefit for selected patients up to 24 hours since last being known well.^6–9^

Provision of thrombectomy is challenging across the NHS because only regional Comprehensive Stroke Centres (CSC) have the necessary facilities and specialist workforce,^10 11^ whereas approximately 70% of patients who had stroke are first admitted to a nearer hospital offering thrombolysis only. When LAO is suspected, rapid transfer to a CSC is necessary to initiate thrombectomy because the chances of a good outcome falls by an average of 6% per 60-minute delay.^7 12^ However, the process of local assessment, interhospital communication and transfer typically requires 2 hours, and further delays are common.^10^ This lengthy care pathway increases service complexity while reducing thrombectomy cost-effectiveness through protracted time to treatment. Delays could potentially be reduced if more patients with LAO were directly admitted to CSCs; however, there is currently no portable diagnostic test or stand-alone LAO symptom assessment with sufficient accuracy to identify the minority of patients with suspected stroke suitable for prehospital redirection^13–15^ and thrombectomy.^9^ A large-scale shift to central admission would decompensate both the CSCs and local hospitals.

A recent service evaluation from Sweden described a novel two-stage approach for identifying a high proportion of patients with LAO in the ambulance and accelerating thrombectomy without high volumes of CSC admissions.^16^ During the first stage, ambulance practitioners use a basic prehospital symptom screening assessment (arm and leg weakness) to trigger the second stage, a telephone communication with a remote CSC specialist who selects patients for direct admission to the CSC if the main clinical criteria for thrombectomy are present. An increase in the volume and speed of thrombectomy treatment was reported, although approximately 50% of admissions to the CSC did not have LAO and 25% of all patients with LAO still required secondary transfer. In the absence of an alternative strategy, it might be possible to adapt this approach to improve thrombectomy delivery in other settings. However, is it unknown whether a two-stage cross-service emergency stroke pathway to improve thrombectomy access is appropriate for NHS services.

Implementation Science literature highlights that the successful development, implementation and adoption of new interventions/pathways is more likely to occur if considered acceptable and feasible by existing services/stakeholders.^17 18^ Therefore, we report on findings from a three-phase, rapid, qualitative study which sought health professionals views about the acceptability, feasibility and the wider impact of a proposed prehospital redirection pathway to select patients for direct admission using a simple ambulance screening trigger followed by remote specialist selection.

## Methods

### Design

This was a deductive, pragmatic multiphase qualitative study using online focus groups/semi-structured interview methods, guided by the Non-adoption, Abandonment and Challenges to Scale-up, Spread and Sustainability (NASSS), Implementation framework.^19^ This is an evidence-based, theory-informed pragmatic framework to understand/predict how an initiative may succeed or fail when implemented. The domains included are the condition/illness, the technology/change, the value proposition, the adopter(s), the organisation(s), the wider (institutional/societal) context and embedding/adaptation over time. The study is reported according to the Consolidated criteria for Reporting Qualitative research (COREQ).^20^

### Participants, sampling and recruitment

Study participants were NHS ambulance paramedics/managers, hospital clinicians/managers and clinical staff from Integrated Stroke Delivery Networks (ISDNs) across England. A sampling frame was used to guide recruitment. Purposive and snowball sampling approaches were used to ensure maximum variation, including region/rurality and seniority. Most participants came from the purposive sampling with two people recruited via recommendation.

The recruitment process was via a contact at each Trust or ISDN, who circulated an email invitation to appropriate staff which included the contact details for the qualitative researchers. Dates/times for focus groups and interviews were arranged flexibly to minimise staff disruption. Written or verbal (recorded via Microsoft Teams) consent was obtained. Participants were offered an optional £30 voucher for each attendance at a focus group/interview.

### Data collection and analysis

We completed three rapid phases of online focus groups and individual/group interviews from January to June 2022. These were conducted by JD and RS and analysed by RS, JD and C Pope and discussed with all coinvestigators. Iterative semi-structured topic guides were used. Initial questions were designed collaboratively by the authors to meet the study aims. The guides explored views on (1) current issues with access to treatment, (2) practicalities of specialist remote assessment, (3) implications for patient safety, (4) possible knock-on effects of changing the pathway, (5) what to include in a remote assessment, (6) acceptable time windows, (7) staff training/information needs and (8) materials to support a change. Digital recordings of focus groups/interviews were transcribed verbatim and anonymised. NVivo Qualitative Software package was used for management/coding of data. We used the framework approach^21^ guided deductively by the NASSS framework.

A coding framework was developed post-interviews, initially using the NASSS domains and refined during analysis. All transcripts were coded to the following domains: the condition, the technology/change, the value proposition, the adopter system, the organisation(s) and the wider (institutional and societal) context plus ‘another’ code to capture non-NASSS interesting data. Key themes were identified/discussed and categorised as ‘simple’ (straightforward, predictable, few components), ‘complicated’ (multiple interacting components or issues) or ‘complex’ (dynamic, unpredictable, not easily disaggregated into constituent components) as defined by NASSS. We supplemented the framework approach with ‘thematic networks’^22^ to identify how dominant/conceptually important themes related to and/or organised lower order themes.

Reliability and validity of the datasets were achieved through intercoder reliability checking of randomly selected transcripts and researcher immersion in the data. ‘Informant’ validation was gained by circulating three reports of interim findings and asking for feedback from study participants.^23^ Triangulation of the findings was achieved through discussions within the research team and study co-investigators—there were no significant disagreements.

### Reflexivity

The qualitative research team (JD—social psychologist, RS—social scientist, C Pope—medical sociologist), all non-clinicians, represented a range of complementary interests, skills and experience. Regular meetings were held by the team to discuss individual and collective academic interests and motivations for involvement in the study.

### Patient and public involvement

A stroke survivor was a study co-investigator and contributed to the review of the study design and supporting study documents.

## Results

### Participants

Twenty-eight participants (table 1) were recruited from nine NHS Trusts (five Hospital Trusts and four Ambulance Trusts) and three ISDNs across England. Four focus groups, 2 group interviews and 18 individual interviews were completed. Nine participants took part in more than one round of data collection. The focus groups lasted between 65 and 90 min and interviews between 35 and 80 min, totalling 23 hours.

**Table 1:** Characteristics of participants by professional role

Ambulance paramedics	Ambulance manager/ISDN	Hospital clinicians	Hospital manager/ISDN
Research paramedic	Lead paramedic critical care	Stroke consultants × 5	Stroke services manager
Paramedic	Consultant paramedics × 3	Consultant in emergency care	Head of services for stroke medicine
Advanced paramedic	Associate medical director	Consultant interventional neuroradiologist	ISDNs × 2
Student paramedic	ISDN	Consultant neurologist	
		Consultant radiologist	
		Stroke specialist nurses × 5	
**Total=4**	**Total=6**	**Total=14**	**Total=4**

ISDN, Integrated Stroke Delivery Network.

### NASSS-informed themes

We identified five themes encompassing data from all three phases of the study: (1) (suspected) stroke as a condition, (2) the pathway change, (3) the value participants placed on the new pathway, (4) the possible impact on NHS organisations and adopter systems, (5) the wider context. These are represented in table 2, with examples of participant quotations to illustrate each theme.

**Table 2:** Deductive NASSS-informed themes and examples of illustrative quotations

Theme 1: (Suspected) stroke as a condition (‘complicated’/‘complex’)	
(Suspected) stroke is a complicated/complex condition: Symptoms can vary and be misunderstood by both ambulance paramedics and the public. Category 2 ambulance response times for stroke—long waits/delays can have adverse outcomes.	“…the way that strokes present is very variable. Obviously you’ve got your classic sort of FAST positive and those ones are the easy ones, aren’t they? But it’s the perhaps the slightly unusual presentations the stroke mimics, the cerebellar strokes. So, the ones that affect the back of the brain or the base of the brain often present with different symptoms and that are quite nonspecific. So, things like Vertigo and vomiting and balance problems and not the classic facial loss, speech and all that kind of stuff that we sort of associate with stroke…And I think that’s an issue from an ambulance response point of view and also from a public perspective. You know, I think there’s still a little bit of misunderstanding from them as to what constitutes the symptoms of a stroke.” (021 Ambulance manager) “So, what we find is a lot of our stroke patients experience long waits so they get called in and they go into our stack of jobs that are waiting for dispatch…and because they tend to fall into either cat two or worse from a stroke patients perspective cat three…We see adverse events for stroke patients who waited for long periods of time and have adverse outcomes.” (023 Ambulance manager)
**Theme 2: The pathway change (‘complicated’)**	
Decision-making: Hospital clinicians face complicated issues.	“…(if) I’m fielding the referrals from a hospital five miles away and a hospital 50 miles away and a 27 year old and a 52 year old and there’s all those different things that you need to take in, where are they? What’s their deficit? Do we think it’s going to be an easy procedure when they get there? Are they at 1 hour so we’ve got a bit of time to play with, so even if they're in an ambulance for another hour, they'll still get here within 2 hours or are they at 3 1/2 hours? And even if it’s a half hour ambulance drive, that means they're going to get here at 4 hours and then you’re starting to ‘I’m not having that’ (because patient is outside time window for treatment) and then the juggle with beds…where are we going to put these people? You know, we can’t have bunk beds…” (027 Hospital clinician)
Use of video or telephone to conduct remote assessment: Mixed views but happy to use either.	“So, we’re using video technology, not in stroke at the moment, but certainly in frailty and some other areas where there’ll be that video consultation because it’s the old saying, a picture paints 1000 words. So, you can talk about a patient on the phone but if you see them in front of you, it gives you a lot more information as a clinician.” (001 Ambulance paramedic)
**Theme 3: The value participants placed on the new pathway (‘complicated’)**	
Mostly positive views Some concerns: Patient/carer experience	“Having seen it from the other side and if we can avoid…where you need that long term rehab, it’s wonderful.” (007 Ambulance paramedic) “I think it’s (new pathway) feasible…The numbers aren’t huge so the knock-on effect for the ambulance service is going to be minimal…The patient experience, like we discussed before, is going to be better.” (019 Ambulance Paramedic) “So, yeah…I think a direct access thrombectomy pathway from my service’s perspective, absolutely the right thing for patients.” (012 Hospital clinician) “…we see the impact it can make…not every case is, you know that spectacular, but the ones where you see an immediate change. That’s what drives you forward…therefore if we can find ways of getting people there quicker.” (016 Hospital clinician) “Stroke is a life changing event…for that person and for that family it’s a massive change…to be able to visit whenever it’s possible to be able to visit within distance, it’s a major issue…we know at least four out of five are patients over age 50, and therefore their companions are also equally older and frail, and you know that travel to a tertiary centre for visiting, for a stroke mimic, for example, is an absolute travesty.” (011 Hospital clinician)
**Theme 4: The possible impact on NHS organisations and adopter systems (‘complicated’)**	
Welfare of ambulance crews	“…we’ve got to think about the welfare of our Crews. You know if they have an hour and a half overrun and they then finish 40/50 miles away from their base station, they’re not coming in the following day. They’re unsafe to drive home and when they do actually get back to base and these issues have resulted in poor outcomes for paramedics when driving home after long overruns.” (014 Ambulance manager)
Who should conduct remote assessment at the CSC: Balance of skills/availability needed.	“From my point of view, having those discussions through a specialist nurse, if they’re not the ones to make the decision to say yes or no to accept, doesn’t feel efficient because I think that we should be speaking directly to another consultant…I think it’s probably important that they speak directly to the person who can give them an answer there and then, now if the specialist nurses at the tertiary centre are trained up to a level of competence or empowered to make those decisions, that’s fine.” (010 Hospital manager)
**Resources:** Lack of space and hospital beds.	“…and then I think another sort of more recent challenge is just the lack of capacity at the hospitals, so physically not having a space to take a patient even when you’ve pre-alerted and they’ve accepted…But yeah, that’s definitely a real issue…” (005 Ambulance paramedic)
Repatriation of patients: Can overwhelm services.	“…and then they either need repatriation or we end up, you know, kind of it. It would overwhelm our emergency department and our stroke physicians. And then we sort of bat this back and forth.” (016 Hospital clinician)
Potential ‘knock-on’ effects on services of new pathway: For CSCs. For stroke units without a thrombectomy service.	“…so if you are looking at the future, you’re looking at all stroke calls within 24 hours or something like that, so that is, in my view, impossible to triage… it’s possible to try it, but actually to get that workforce to try it and do nothing else, is I think, not sustainable…I think you would also need an extra layer of workforce to actually do extended thrombectomy because those patients now that are just getting aspirin and going to the ward will now have CTA CTP discussion with radiology. So, we’ll take less number of patients, but we’ll take longer…to fund another group of people to run a 24/7 ambulance triage is not a possible solution from a funding and business case point of view.” (002 Hospital clinician) “So, they would lose some of their hyper acute patients who you know, the other hospitals would lose some of their hyper acute patients, which will obviously impact on the number of admissions they are having and services they provide and for the resources they get for that.” (002 Hospital clinician)
**Theme 5: The wider context (‘complicated’)**	
Regional variations in services and stroke pathways/protocols	“One of the things I’d love to see is a standardised national ‘this is what we do’ that would make life so much easier for the paramedics.” (001 Ambulance paramedic)
Lack of standardisation and variable connectivity in information and communications technology	“…it’s the actual system to support what you want…and that’s then relying across different hospital trusts and different organisations having access to the same system.” (012 Ambulance paramedic)
Staffing levels/demands	“…on the radiologist side, the radiographer side and the nursing side in in all three groups we are struggling to recruit…we’ve got problems acquiring the images and interpreting the images…” (017 Hospital clinician) “I know often the challenge is increasingly getting them within the window, especially as demands going up, and I will trust the feeling that we’re not getting to patients as quickly as we’d like to be…and I think that we’re, you know, we’re often arriving a couple of hours into the call, and so the actual the window to get them to a HASU is becoming more challenging.” (005 Ambulance clinician)

NASSS, Non-adoption, Abandonment and Challenges to Scale-up, Spread and Sustainability, Implementation.

### Theme 1: the condition (suspected) stroke

This theme describes how the complex nature of (suspected) stroke would need to be considered during the development and implementation of an effective new thrombectomy pathway. Issues discussed included symptom recognition prediagnosis; barriers to accessing timely treatment such as difficulty in establishing onset time; and how the current ambulance dispatch response time categorisation might delay time-critical treatments. Some participants believed the ambulance response should be upgraded to the same as suspected cardiac arrest (Category 1)^24^ to maximise the value of any new stroke pathway.

Participants also discussed how patient factors could create barriers to seeking help when stroke occurs and reduce the potential value of a new thrombectomy pathway. For example, despite a long running national public awareness campaign in the UK about contacting emergency medical services if common stroke symptoms occur, younger people and members of some minority ethnic groups may not be sufficiently aware and therefore generally reluctant to seek medical help.

### Theme 2: the pathway change

This theme explores participant views on the components of a potential pathway: the use of FAST (Face, Arm, Speech, Time) as a potential screening tool by ambulance personnel; time ‘windows’ for thrombectomy treatment; and the use of telephone and/or video for remote assessment with stroke specialists.

Mixed views were expressed about the potential use of FAST as the main paramedic assessment tool for suspected stroke. FAST alone was not generally considered as suitable for identifying patients needing a thrombectomy. Suggestions were made for more sophisticated assessment tools and decision-making aides to enhance the selection process when used independently; however, participants were mindful of balancing time spent assessing the patient with the need for a ‘slick and robust’ time efficient approach and trade-offs for sensitivity and specificity.

The time ‘window’ used to select patients for redirection to the CSC stimulated discussion on whether this should be fixed or patient-centred and nuanced. Opinions were divided on this issue with some clinicians concerned that a fixed time window might disadvantage some patients still suitable for thrombectomy up to 24 hours.^8^ Other stroke clinicians preferred the certainty of a fixed time window if being used by non-specialists in the prehospital setting. This range of views appeared to reflect differences in clinician interpretation of the emerging evidence describing which patients could potentially benefit from thrombectomy, but also the degree to which they believe their own services could cope with the disruption created by a new direct admission pathway.

There were mixed views on the use of telephone or video for a remote specialist thrombectomy assessment with pros and cons given for both methods. Patchy connectivity was cited as a possible problem with using live video together with variable signal strengths for mobile phones, particularly in rural areas. While some clinicians had direct experience of using video and outlined some drawbacks, others felt seeing a patient could improve the assessment and exclude unnecessary redirection of patients. Overall, participants were happy to use either method for a remote assessment if it was readily available and avoided prehospital delays.

### Theme 3: the value participants placed on the new pathway

This theme explores how participants perceived the potential value of the proposed pathway. Participants agreed about the benefits of thrombectomy and were mostly in favour of the proposed redirection pathway. They believed this to be the right approach for patients with LAO to access treatment quickly, thereby reducing deaths, permanent disablement and the need for costly long-term rehabilitation.

In contrast, a concern was expressed about how the proposed pathway could affect patients’ experience of care especially if transferred to a CSC at some distance from their home. Increased travel time for visiting families/friends was viewed, by one participant, as being detrimental. A possible risk to patient safety was raised if the proposed pathway increased the numbers of patients needing specialist hospital treatment/equipment, potentially leading to ‘log jams’, unintended treatment delays and poorer clinical outcomes. Furthermore, a longer ‘on-scene’ time for screening/assessment could pose a risk (through delayed treatment) for patients assessed as unsuitable for a thrombectomy.

### Theme 4: the possible impact on NHS organisations and adopter systems

This theme relates to the complicated configuration of organisations/services deployed along the existing stroke pathways and the possible impact of the proposed pathway. The main challenges relate to the ‘knock-on effects’ of the pathway on the routine functioning of ambulance services, thrombectomy and non-thrombectomy hospitals and specialist departments. Key issues identified were staffing levels/burn out, access to scanning/imaging equipment, increased/decreased workflow distracting from key care delivery goals and maintenance of a specialist workforce, funding and bed capacity.

Organisational resources were discussed in some depth and participants did not perceive the new pathway as an ‘existential threat’ to NHS organisations/services. Possible dilemmas were explored, such as who should/could conduct the remote assessment at the CSC. To avoid overloading stroke specialists, some participants argued that trained stroke nurses could perform this role while others thought that the person taking the call needed to be the thrombectomy ‘decision maker’, normally a consultant.

Education/training to deliver the proposed new pathway was discussed throughout the study. A lack of training time for paramedics was highlighted and concerns raised over any significant additional training to deliver a new pathway. For effective implementation, it would be important for the pathway to easily integrate into existing standard care with minimal need for new skills/knowledge. Nearly all participants agreed that ongoing feedback to ambulance paramedics was important for professional/service development and confidence building, but this is not easily facilitated without additional resources. The proposed pathway might mean a cultural change for some paramedics if it is shown that longer assessment and transfer times can in fact improve outcomes.

Secondary transfers or ‘repatriation’ of patients was also noted as a potential challenge which could result in lengthy displacement periods for patients and corresponding pressures on hospital beds.

### Theme 5: the wider context

This theme explores the wider current socio/cultural/medical context and the impact on health staff and services. Participants discussed how the COVID-19 pandemic has taken its toll on health services resulting in staff shortages, staff burnout, hospital bed shortages and longer ambulance response times due to increased demand, staff sickness and delays at hospitals.

Regional geographical variations in ambulance and hospital services, stroke pathways, protocols and admission criteria were discussed in depth. These factors influenced staff perceptions of the local viability of the proposed pathway. The geography and proportion of rurality/urban characteristics of a region can result in ambulance crews working ‘out of area’ and face challenges in navigating regional variations in stroke protocols.

Concerns were raised about a current lack of 24/7 access to CSCs across the NHS, and a paucity of imaging equipment alongside a current national shortage of staff to interpret diagnostic images (notably perfusion imaging). This situation could be aggravated if the proposed pathway generates increased demand and is implemented before services have the capacity to offer better care to a larger number of directly admitted patients. Paradoxically, without this increased demand these factors may not be prioritised for improvement, and the pathway could enable concentration of patients at sites with optimal facilities and specialist workforce provision so that centralisation of resources becomes a more attractive possibility.

## Discussion

We conducted a pragmatic, multiphase, qualitative research study informed by NASSS to describe multiprofessional and regional health service personnel views on the acceptability/feasibility of a new two-stage direct admission pathway for thrombectomy. Our analysis suggests that suspected stroke is already a ‘complicated’ and, in some cases, ‘complex’ scenario.^19^ Participants consistently supported the concept of a thrombectomy pathway, which they felt was likely to bring significant benefits for selected patients but cautioned regarding the perceived realities of implementing the pathway within a complex mixture of cultural factors, changing patient demographics, variable regional healthcare provider systems, time-dependent treatment factors and a challenging sociopolitical healthcare context. Understanding this context is important for overcoming challenges in efficient thrombectomy provision, which remains a time-critical treatment with limited availability.^18^

Participants expressed positive views about the rationale and value of using a simple prehospital trigger plus remote specialist selection as an acceptable approach for early identification of patients potentially suitable for thrombectomy. However, they recognised that this approach would not operate with full efficiency because both ambulance and hospital services cannot always respond promptly due to finite resources, and some patients will later be found to have a different diagnosis. There were uncertainties about whether the CSC call taker should be an experienced nurse or specialist, and whether video would have advantages over telephone review. As it is unclear whether any of these options would be advantageous, the new pathway should be examined in different settings to evaluate the most effective components. Participants identified challenges around possible ‘knock-on effects’ on existing services although these seemed more evident for hospital stroke centres than ambulance services. There was particular unease about increased demand on bed capacity at CSCs if timely repatriations of both appropriately and inappropriately redirected patients were not achieved. Therefore, in parallel with pathway implementation, we suggest there should be processes in place for rapid return to local hospital settings, which has previously been demonstrated as safe within 6–12 hours post-thrombectomy for selected patients.^25^

Ambulance service participants voiced frustration about existing variations in processes for admitting stroke patients and were supportive of attempts to create standardised evidence-based emergency pathways. Participants argued that there are few situations with such strong evidence that a time-critical treatment reduces future disability and care costs for a common condition, and that re-categorisation to the most urgent category would be beneficial for maximising the value of a new pathway. However, there were concerns that stroke centres without a thrombectomy service may experience a reduction in activity and resources if a new pathway reduces local demand. Culture shifts would also be required for both hospital (more dynamic, fast-paced, interdisciplinary and co-operative approach) and ambulance (possible longer on-scene/transfer times to make a phone/video call to a CSC) personnel. Demographic factors were highlighted, such as age and ethnicity, which could impact on the value of the proposed pathway through poor symptom awareness, and it may be necessary to provide additional targeted public education through appropriate channels.

The results from our study echo previous findings from evaluations of the implementation of thrombectomy and telemedicine in emergency stroke care.^12 26–28^ Improving treatment times was highly valued but it was acknowledged that challenges include safety, demand, logistics/timing, workforce education/culture, variability of stroke services, service/professional cooperation and information/communication technology connectivity/rurality. The findings also overlapped with experiences reported by professionals regarding direct admission policies to specialist centres for other prehospital scenarios, notably myocardial infarction and major trauma. When centralised primary angioplasty services were first established, staff welcomed how multidisciplinary specialist pathways provided efficient care but also emphasised the need for cross-organisational planning and training to ensure effective implementation and equitable access.^29 30^ Despite these initial concerns, mixed methods evaluation of redirection for selected patients with myocardial infarction has demonstrated better outcomes, good use of healthcare resources and acceptability to staff and patients and therefore remains the standard model for providing emergency primary angioplasty.^30^

Following the introduction of pathways to select casualties for immediate transportation to regional trauma centres, professionals highlighted the importance of standardising initial assessment to identify appropriate individuals, while raising concerns about the possible de-skilling of local hospitals^31–33^ and the subsequent challenges for local repatriation. Despite concerns, this model was associated with significant improvements in both the care process and outcomes of patients after severe injury.^33^ However, emergency stroke care also has important differences to these scenarios which justify specific examination of how best to optimise thrombectomy access for remote populations, such as less certainty about the initial diagnosis (making unnecessary transfer more of a possibility); a higher frequency of co-morbidities likely to impact on treatment decisions; the sizeable proportion of patients who require multidisciplinary care over a longer timeframe (rather than a short-term high-impact medical intervention) and the ongoing evolution of thrombectomy service infrastructure within hospitals. Successful implementation of a new pathway will first require careful consideration of these wider issues and the related population level trade-offs in appropriately designed clinical trials.

### Limitations

We gained a range of ambulance and hospital professionals views with varying lengths of experience from four regions in England. Although the regions were representative of services where the pathway would be most relevant and disruptive if implemented, we were unable to obtain views across all regions and although some perspectives around demographic diversity were gained; this could be explored further. Data collection was undertaken during a time of exceptional pressure on NHS personnel/services which could have affected the views of participants.

## Conclusions

This qualitative study gave ‘voice’ to generally positive views of ambulance and hospital personnel on a two-stage prehospital redirection pathway which would combine an ambulance trigger with a remote specialist assessment. Concerns were expressed about multiple factors which could limit implementation, plus possible negative effects such as greater patient flow to CSCs causing pressure on capacity and demand for repatriation. Real-world evidence is needed to describe pathway impact on thrombectomy provision, experiences of the wider suspected stroke population and consequences for services and professionals.

## Data Availability

All data relevant to the study are included in the article or uploaded as supplementary information.
